# High Throughput Molecular Confirmation of β-Thalassemia Mutations Using Novel TaqMan Probes

**DOI:** 10.3390/s130202506

**Published:** 2013-02-18

**Authors:** Siew Leng Kho, Kek Heng Chua, Elizabeth George, Jin Ai Mary Anne Tan

**Affiliations:** 1 Department of Biomedical Science, Faculty of Medicine, University of Malaya, Kuala Lumpur 50603, Malaysia; E-Mails: khosiewleng@yahoo.com (S.L.K.); khchua@um.edu.my (K.H.C.); 2 Department of Pathology-Hematology, Faculty of Medicine and Health Sciences, University Putra Malaysia, Serdang, Selangor 43400, Malaysia; E-Mail: elzageorge@hotmail.com

**Keywords:** β-thalassemia, Malaysia, Malay, quantitative real-time PCR, TaqMan

## Abstract

β-Thalassemia is a public health problem where 4.5% of Malaysians are β-thalassemia carriers. The genetic disorder is caused by defects in the β-globin gene complex which lead to reduced or complete absence of β-globin chain synthesis. Five TaqMan genotyping assays were designed and developed to detect the common β-thalassemia mutations in Malaysian Malays. The assays were evaluated with 219 “blinded” DNA samples and the results showed 100% sensitivity and specificity. The in-house designed TaqMan genotyping assays were found to be cost- and time-effective for characterization of β-thalassemia mutations in the Malaysian population.

## 1. Introduction

Malaysia is a multi-racial country with a population of around 28 million. The Malays form the largest ethnic group and make up around 53.5% of the population. β-Thalassemia is a public health problem in Malaysia and approximately 4.5% of the Malays and Chinese are β-thalassemia carriers [1]. β-Thalassemia is mainly caused by point mutations and small deletions which lead to reduced (β^+^) or absence (β°) of β-globin chain synthesis. Beta-thalassemia major patients are unable to survive into adulthood without life-long monthly blood transfusions and iron chelation therapies [2].

Since 1990, the amplification refractory mutation system (ARMS) has been widely used in β-thalassemia mutation detection with two primers that are complementary to the normal and mutant DNA sequences, respectively [3]. Although this technique is sensitive in mutation detection, ARMS can be time-consuming due to the many assays that need to be carried out for detection of different mutations. Restriction fragment length polymorphism and reverse dot-blot hybridization have also been used in mutation characterization, but these techniques can also be time-consuming [4,5]. Another approach, quantitative real-time PCR (qPCR) using saturated dye coupled with high resolution melt-curve analysis has been used for characterization of thalassemia mutations [6,7]. However, different mutations may produce similar melting curves or very small differences in melting temperatures that lead to ambiguous results. Probe-based assays coupled with melting curve analysis are still necessary to deliver results with superior accuracy [8,9].

Rapid and accurate molecular assays are essential for characterization of β-thalassemia mutations in affected families. Sensitive and specific prenatal diagnosis allows couples at risk of producing a β-thalassemia major child to make informed decisions with regard to affected pregnancies. Therefore, this study aims to develop a rapid, sensitive and cost-effective approach for characterization of β-thalassemia mutations using custom-made TaqMan genotyping as the selected platform. Five TaqMan genotyping assays targeting IVS1-1 (HBB:c.92+1G>T), IVS1-5 (HBB:c.92+5G>C), CD41/42 (HBB:c.127_130delCTTT), Poly A (HBB:c.*+112A>G) and CD26 (HBB:c.79G>A) mutations were developed to detect the common β-thalassemia mutations in the Malaysian Malay population. These mutations are responsible for 81% of β-thalassemia in this ethnic group [10,11].

## 2. Experimental Section

### 2.1. TaqMan Genotyping Materials

TaqMan GTXpress™ Master Mix which contains PCR buffer, AmpliTaq Gold DNA Polymerase, deoxyrinucleotides triphosphates (dNTPs) and 6-carboxy-X-rhodamine (ROX) passive reference dye was purchased from Applied Biosystems (Foster City, CA, USA). TaqMan genotyping assays were specifically customized to detect the five common β-thalassemia mutations in Malaysian Malays. Initially, the β-globin gene sequence was obtained from GenBank, followed by thorough primer and probe design using the Primer Express software. The primer sequences constructed were further tested using *in silico* PCR to ensure that they were targeted to the exact loci [12,13]. The TaqMan genotyping assay (40X) contained 36 μM forward primer, 36 μM reverse primer and two probes (8 μM for each probe). The forward and reverse primers were designed to specifically amplify the regions where the mutations are located (Table 1). Two probes were designed for each mutation: one for hybridization to the normal DNA sequence and the second probe for hybridization to DNAwiththemutation (Table 2). The probes were designed with minor groove binder (MGB) and non-fluorescent quencher (NFQ) at the 3′ end, whereas the 5′ end contained the fluorescence reporter dyes 2′-chloro-7′-phenyl-1, 4-dichloro-6-carboxyfluorescein (VIC) or 6-carboxyfluorescein (FAM).

### 2.2. DNA Extraction

Blood samples were obtained from β-thalassemia major patients (n = 21), β-thalassemia carriers (n = 74) and normal individuals (n = 25) with written consent. This study was approved by the Medical Ethics Committee of University Malaya Medical Centre in accordance with the Declaration of Helsinki. Human genomic DNA was extracted from blood samples using a simplified phenol-chloroform method [14]. The DNA samples were diluted to 20 ng·μL^−1^ and used as templates for qPCR. DNA containing the five β-globin gene mutations used for development of the TaqMan genotyping assays was previously characterized by established techniques—ARMS, RFLP, gap-PCR and genomic sequencing.

### 2.3. Development of TaqMan Genotyping Assays

Ninety-five characterized DNA and 25 normal control DNA samples were used in the assay development (Table 3).

Real-time PCR was performed in a real-time thermal cycler (Applied Biosystems 7500 Fast). Cycling conditions involved an initial cycle at 95 °C (20 seconds), followed by 40 cycles of denaturation at 95 °C (3 seconds) and annealing/extension at 60 °C (30 seconds). The final reaction mixture was 10 μL which consisted of 5 μL TaqMan GTXpress™ Master Mix, 0.5 μL TaqMan genotyping assay (20X), 1 μL DNA sample and 3.5 μL double distilled water.

### 2.4. Evaluation of TaqMan Genotyping Assays

Validation was carried out using 219 “blinded” DNA samples in order to determine the sensitivity and specificity of the TaqMan genotyping assays. The 219 “blinded” DNA samples were from 35 normal individuals and 184 previously characterized β-thalassemia patients.

## 3. Results and Discussion

### 3.1. Development of TaqMan Genotyping Assays

**Figure 1.**
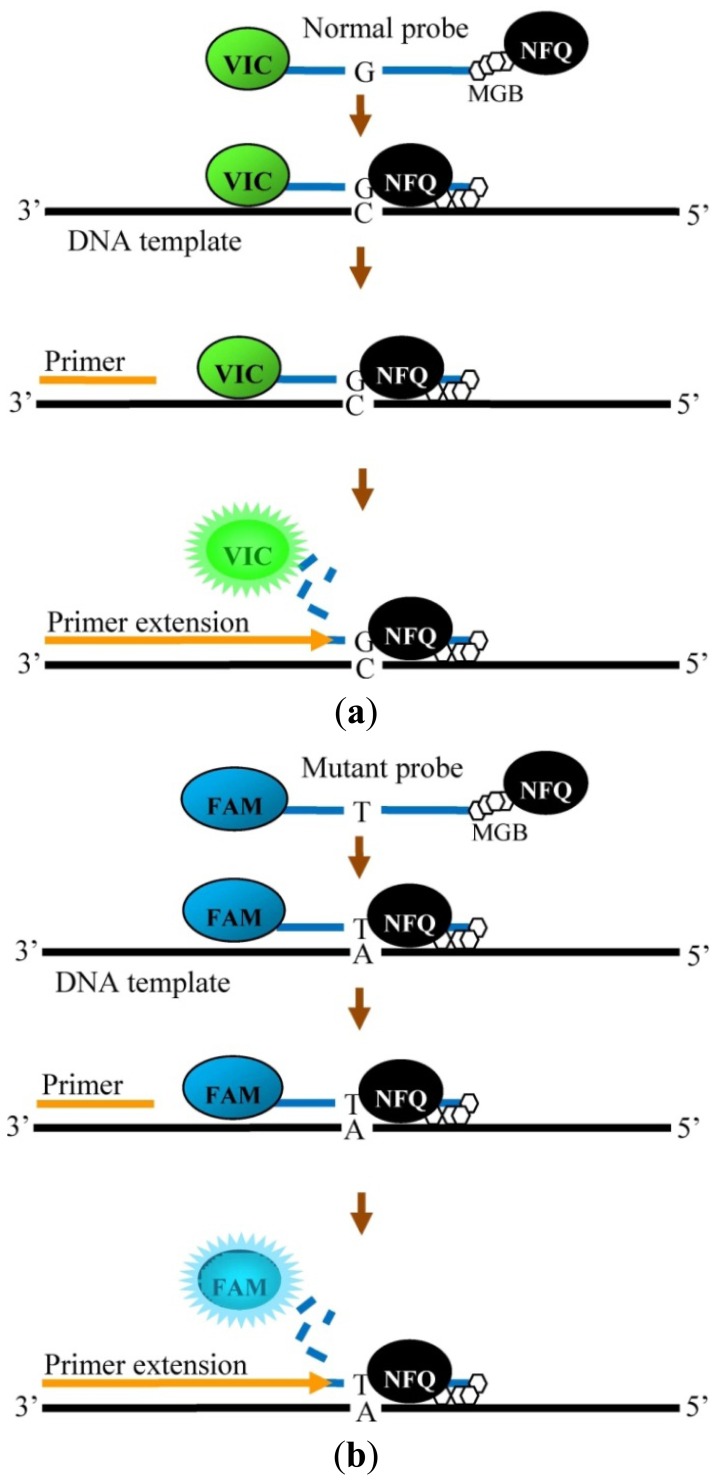
Binding of primers and probes on the DNA template for detection of IVS1-1 (G-T). (**a**) Normal probe of IVS1-1 and primer hybridized to the DNA template. Fluorescence signal is released from VIC dye (green) when cleaved by *Taq* DNA polymerase. (**b**) Mutant probe of IVS1-1 and primer hybridized to the DNA template. Fluorescence signal is released from FAM dye (blue) when cleaved by *Taq* DNA polymerase.

Five TaqMan genotyping assays were designed and established in this study to detect the five common β-thalassemia mutations in Malaysian Malays. The established assays are different from previously developed assays from other countries due to the different mutations in the Malaysian population [8,9]. For our assays, similar forward and reverse primer sequences were used in the IVS1-1 and IVS1-5 genotyping assays as the locationof both mutations are very close to each other. Different primer pairs were designed for the amplification of CD41/42, Poly A and CD26. Specific probes were designed for each mutation to allow precise hybridization with the normal and mutant DNA sequences. The probes were specifically designed with lower annealing temperatures compared with the primers to allow probe binding before primer binding. The 3′ end of the probe contained the NFQ which quenched the fluorescence dye at the 5′ end. In addition, the probes were particularly designed with a MGB at the 3′ end which fitted into the minor groove of duplex DNA. This additional feature in the probe construction allows enhanced stabilization during probe annealing [15]. Figure 1 shows the binding of the primers and probes using IVS1-1 mutation as an example. The final step in the TaqMan genotyping assay involves the cleaving of the probe by *Taq* DNA polymerase followed by release of the fluorescent signal by the fluorescent dye [16].

**Figure 2.**
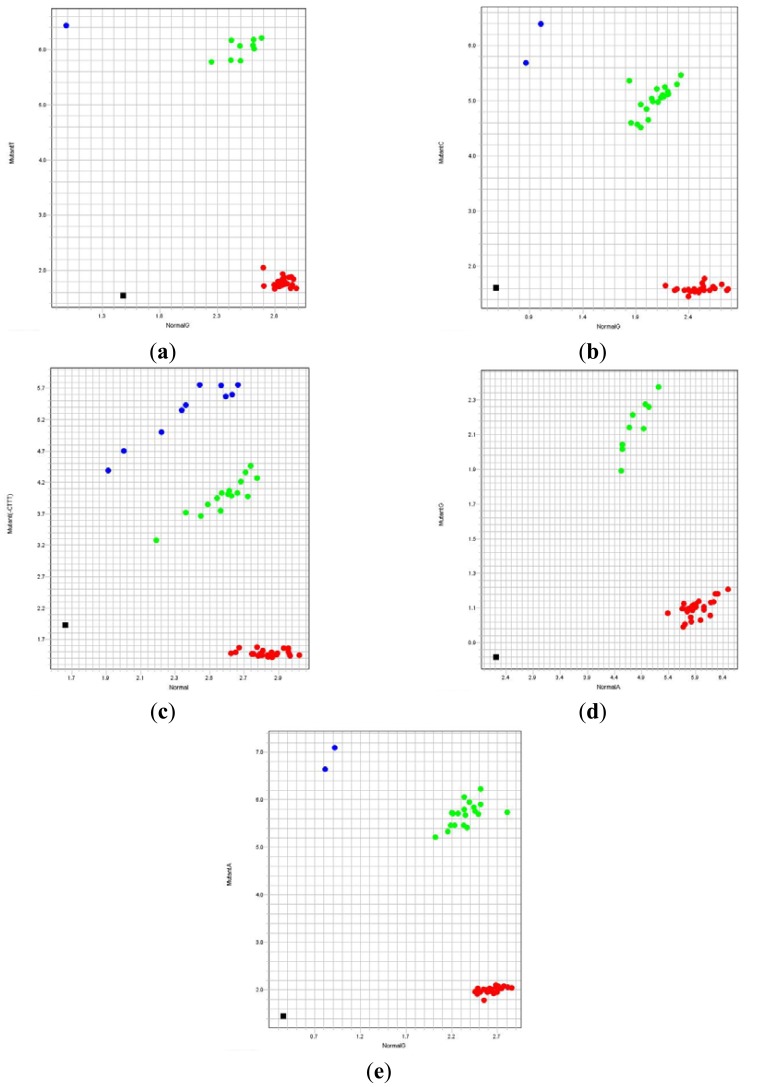
Allelic discrimination plots of TaqMan genotyping assays for the five β-thalassemia mutations; normal allele at x-axis and mutant allele at y-axis. Black dots indicate no amplification (non-template PCR control), red dots indicate individuals negative for the mutation, green dots indicate individuals heterozygous for the mutation and blue dots indicate individuals homozygous for the mutation. (**a**) IVS1-1. (**b**) IVS1-5. (**c**) CD41/42. (**d**) Poly A. (**e**) CD26 (HbE).

The accuracy of the newly designed TaqMan genotyping assays for β-thalassemia was assessed using 120 previously characterized DNA samples. The results were analyzed from the allelic discrimination plots generated by 7500 Software v2.0.6 (Applied Biosystems, Figure 2). The allelic discrimination plot for a particular mutation will show four regions which indicate no amplification (non-template PCR control), individuals without the specific mutation, individuals heterozygous for the mutation or individuals homozygous for the mutation. The developed TaqMan genotyping assays were able to successfully characterize the β-thalassemia mutations in all the 120 known DNA samples.

### 3.2. Evaluation of the TaqMan Genotyping Assays

**Figure 3.**
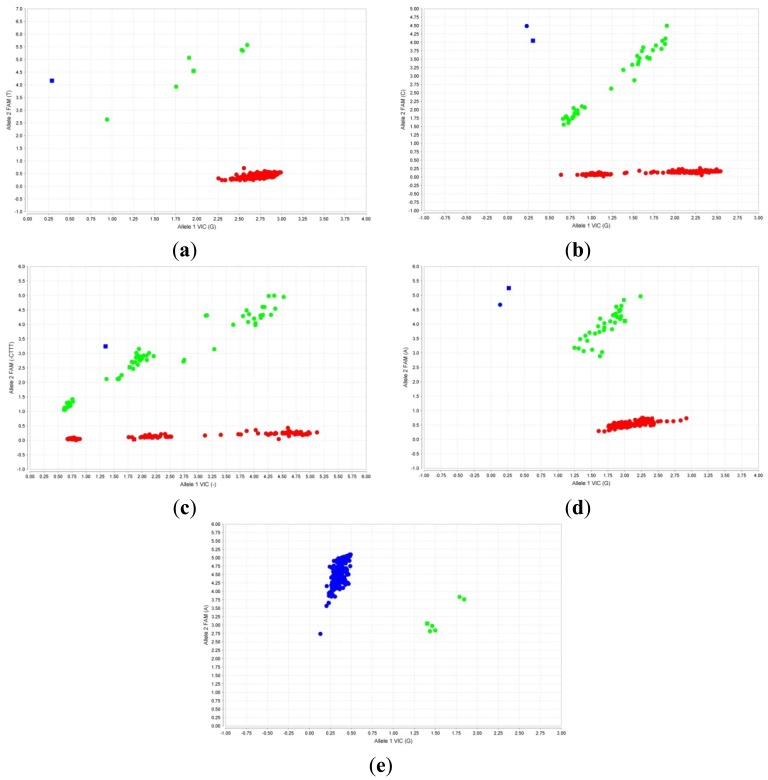
Scatter plots of TaqMan genotyping assays for the 5 mutations with VIC dye fluorescence at x-axis and FAM dye fluorescence at y-axis. Squares indicated controls. (**a**) IVS1-1, (**b**) IVS1-5, (**c**) CD41/42 and (**d**) CD26 (HbE), red dots indicate individuals who do not possess the specific mutation, green dots indicate carriers and blue dots indicate individuals homozygous for the mutation; (**e**) Poly A, blue dots indicate individuals who do not possess the Poly A mutation and green dots indicate carriers.

The in-house designed assays were evaluated by carrying out qPCR using 219 “blinded” previously characterized DNA samples. The results obtained were analyzed using the TaqMan Genotyper Software v1.2 (Applied Biosystems). Characterized DNA samples were also used as controls for each assay. The fluorescent signals for each of the five β-thalassemia mutations were plotted in the scatter plot as shown in Figure 3. The results showed 100% concordance with results previously obtained using the established ARMS technique. This clearly indicates that the newly designed TaqMan genotyping assays produce 100% sensitivity and specificity for the five common Malay β-thalassemia mutations.

In summary, the TaqMan genotyping assays only require a single qPCR to differentiate between normal, carriers and individuals homozygous for a mutation, whereas ARMS requires tworeactions - DNA amplification for normal sequence and another reaction for mutant sequence. Thus, ARMS is more time-consuming and even though multiplex-ARMS has been developed, this assay has its limitation of non-specificity. Since 1 μL of DNA with concentration of 20 ng·μL^−1^ is required in qPCR, whereas 1 μL of DNA with concentration of 1 ng·μL^−1^ is required in ARMS, qPCR using TaqMan genotyping assay is a more valuable assay for prenatal diagnosis and preimplantation diagnosis. The rapidity and cost-effectiveness of the TaqMan genotyping assays will also allow for high throughput screening of β-thalassemia mutations.

## 4. Conclusions

Five TaqMan genotyping assays which allow rapid confirmation of common thalassaemia in Malaysian Malays were developed in this study. The assays showed 100% sensitivity and specificity. The system is reproducible and time-effective. Large-scale β-thalassemia mutation screening together with genetic counseling will certainly lead to a reduction in the birth of β-thalassemia major children.

## Figures and Tables

**Table 1.** Primer sequences for DNA amplification of the five common β-thalassaemia mutations in the Malaysian Malay population.

**Mutation**	**Amplicon Size (bp)**	**Forward Primer Sequence (5′-3′)**	**Reverse Primer Sequence(5′-3′)**
**IVS1-1(G-T)IVS1-5(G-C)**	90	GGTGAACGTGGATGAAGTTGGT	GCCCAGTTTCTATTGGTCTCCTTAA
**CD41/42(-CTTT)**	69	GCTGGTGGTCTACCCTTGGA	ACAGCATCAGGAGTGGACAGATC
**Poly A(A-G)**	96	GGGCCTTGAGCATCTGGATT	CCCACATTCCCTTTTTAGTAAAATATTCAGAAATAAT
**CD26 (HbE)(G-A)**	79	GCAAGGTGAACGTGGATGAA	GTCTCCTTAAACCTGTCTTGTAACCT

**Table 2.** DNA sequences of the normal and mutant probes for TaqMan genotyping of the five common β-thalassaemia mutations in Malaysian Malays.

**Mutation**	**Normal probe (5′-3′)**	**Mutant probe (5′-3′)**
**IVS1-1(G-T)**	VIC-CTGGGCAG 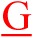 TTGGTAT-MGB-NFQ	FAM-CTGGGCAG 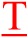 TTGGTAT-MGB-NFQ
**IVS1-5(G-C)**	VIC-CAGGTTG 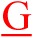 TATCAAGG-MGB-NFQ	FAM-CAGGTTG 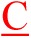 TATCAAGG-MGB-NFQ
**CD41/42(-CTTT)**	VIC-CCAGAGGTT 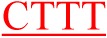 GAGTC-MGB-NFQ	FAM-CAGAGGTTGAGTCCT-MGB-NFQ
**Poly A(A-G)**	FAM-TGCCTAATA 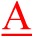 AAAACA-MGB-NFQ	VIC-CTGCCTAATA 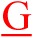 AAAACA-MGB-NFQ
**CD26 (HbE)(G-A)**	VIC-TGGTGGT 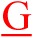 AGGCCCT-MGB-NFQ	FAM-TTGGTGGT 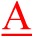 AGGCCCT-MGB-NFQ

The differences between normal and mutant probes are indicated in red.

**Table 3.** List of DNA samples from individuals who are compound heterozygous, homozygous and heterozygous for β-thalassaemia.

**Compound Heterozygotes**		**Homozygotes**		**Heterozygotes**	
		
**Mutation**	**Number**	**Mutation**	**Number**	**Mutation**	**Number**
IVS1-1 and CD26	2	IVS1-1	1	IVS1-1	8
IVS1-1 and CD41/42	1	IVS1-5	2	IVS1-5	19
IVS1-5 and CD26	1	CD41/42	10	CD41/42	19
CD41/42 and CD26	1	CD26	2	Poly A	8
CD41/42 and Poly A	1			CD26	20
Total	6		15		74
